# The phage T4 DNA ligase in vivo improves the survival-coupled bacterial mutagenesis

**DOI:** 10.1186/s12934-019-1160-7

**Published:** 2019-06-13

**Authors:** Junshu Wang, Fapeng Liu, Tianyuan Su, Yizhao Chang, Qi Guo, Qian Wang, Quanfeng Liang, Qingsheng Qi

**Affiliations:** 1 State Key Laboratory of Microbial Technology, Shandong University, Qingdao, 266237 People’s Republic of China; 2 National Glycoengineering Center, Shandong University, Qingdao, 266237 China; 3 CAS Key Lab of Biobased Materials, Qingdao Institute of Bioenergy and Bioprocess Technology, Chinese Academy of Sciences, Qingdao, 2566101 China

**Keywords:** T4 DNA ligase, Microbial mutagenesis, Atmospheric and room temperature plasma (ARTP), Polyhydroxybutyrate (PHB)

## Abstract

**Background:**

Microbial mutagenesis is an important avenue to acquire microbial strains with desirable traits for industry application. However, mutagens either chemical or physical used often leads narrow library pool due to high lethal rate. The T4 DNA ligase is one of the most widely utilized enzymes in modern molecular biology. Its contribution to repair chromosomal DNA damages, therefore cell survival during mutagenesis will be discussed.

**Results:**

Expression of T4 DNA ligase in vivo could substantially increase cell survival to ionizing radiation in multiple species. A T4 mediated survival-coupled mutagenesis approach was proposed. When polyhydroxybutyrate (PHB)-producing *E*. *coli* with T4 DNA ligase expressed in vivo was subjected to ionizing radiation, mutants with improved PHB production were acquired quickly owing to a large viable mutant library generated. Draft genome sequence analysis showed that the mutants obtained possess not only single nucleotide variation (SNV) but also DNA fragment deletion, indicating that T4 DNA ligase in vivo may contribute to the repair of DNA double strand breaks.

**Conclusions:**

Expression of T4 DNA ligase in vivo could notably enhance microbial survival to excess chromosomal damages caused by various mutagens. Potential application of T4 DNA ligase in microbial mutagenesis was explored by mutating and screening PHB producing *E. coli* XLPHB strain. When applied to atmospheric and room temperature plasma (ARTP) microbial mutagenesis, large survival pool was obtained. Mutants available for subsequent screening for desirable features. The use of T4 DNA ligase we were able to quickly improve the PHB production by generating a larger viable mutants pool. This method is a universal strategy can be employed in wide range of bacteria. It indicated that traditional random mutagenesis became more powerful in combine with modern genetic molecular biology and has exciting prospect.

**Electronic supplementary material:**

The online version of this article (10.1186/s12934-019-1160-7) contains supplementary material, which is available to authorized users.

## Background

In nature, mutation combining with natural selection is the key driving force for the life evolution [1]. However, if we would like to generate microbes/organisms with desired properties, the natural evolution process is always not efficient enough and takes long time due to the low spontaneous mutation rates [2–4]. Therefore, varieties of mutagens either physical or chemicals such as ionizing radiation, UV radiation, alkylating agents and azides than may increase the random mutagenesis rate of the target organisms was employed in the laboratory, which provides a way to improve the mutagenesis and evolution efficiency [5].

Both chemical and physical mutagen directly or indirectly causes DNA damage, which leads to the mutation of cell genetic material. Many DNA damage response systems, such as the SOS response, is critical in mediating DNA damage repair. However, these repairing process also tend to generate mutation [6–8]. High dose of the mutagen could produce more DNA damage in cells, which in turn generate more mutations. However, excessive DNA damages are beyond the ability to repair cells within the mechanism that is lethal to cells. In microbial mutation breeding, the rate of lethality in the treated microbes is always more than 90% [9]. Since only viable cells can contribute to a mutation library, improving the cell survival during the mutagenesis process can greatly increase the efficiency of random mutagenesis.

With the development of molecular biology, system biology and synthetic biology, rational modification of microbes to obtain desired properties became fashionable and efficient [10–12]. Endogenous genes were up or down regulated to redirect the metabolic flow to desired routes, while exogenous genes were adopted to obtain the properties that was not existed in the host [13–16]. In the meantime, many approaches were developed to alter the protein expression of metabolic pathway by changing the promoter strength, enzyme stability, or even protein properties [13, 17, 18]. In this case, rational design is prevailing and efficiency are greatly improved in compare to traditional mutagenesis methods. However, the rational design is hampered greatly by current limited knowledge of complicated life and limited ability to interpret complicated cellular networks.

The T4 DNA ligase from Enterobacteria phage has been widely used in molecular biology applications in vitro given its ability of joining both sticky and blunt ended DNA [19]. In addition, T4 DNA ligase is a versatile enzyme capable of catalyzing reactions such as DNA ends relaxation [19]; duplex DNA gap sealing [20]; ligation of DNA with base pair mismatched [21]; nick-closing [22] and oligomerization of bacteriophage [23, 24].

Previous studies have shown that DNA ligase contributes to chromosome DNA nicks repair after in vivo scission by restriction endonuclease [25, 26]. In our recent study, we found T4 DNA ligase in vivo was able to mediate the repair of DNA double strands breaks (DSBs) (Manuscript accepted). This not only increased the survival of the host but also increased the mutation if we treated the microbes with traditional DSBs causing method, for example, random mutagenesis machine atmospheric and room temperature plasma (ARTP-a microbial mutagenesis machine that causes DNA sequences changes). Based on this, a new method called T4 mediated survival-coupled mutagenesis (T4SM) was proposed, which combined the traditional mutagenesis and molecular biology method by in vivo expression T4 DNA ligase to improve the mutagenesis survival of the host. The efficiency of the method was demonstrated using a PHB-producing *E. coli* strain. The obtained mutants were sequenced to gain an understanding between phenotypes and genomic variations.

## Results and discussion

### T4 DNA ligase in vivo increases cell survival to genotoxic drug treatment

**Fig. 1:**
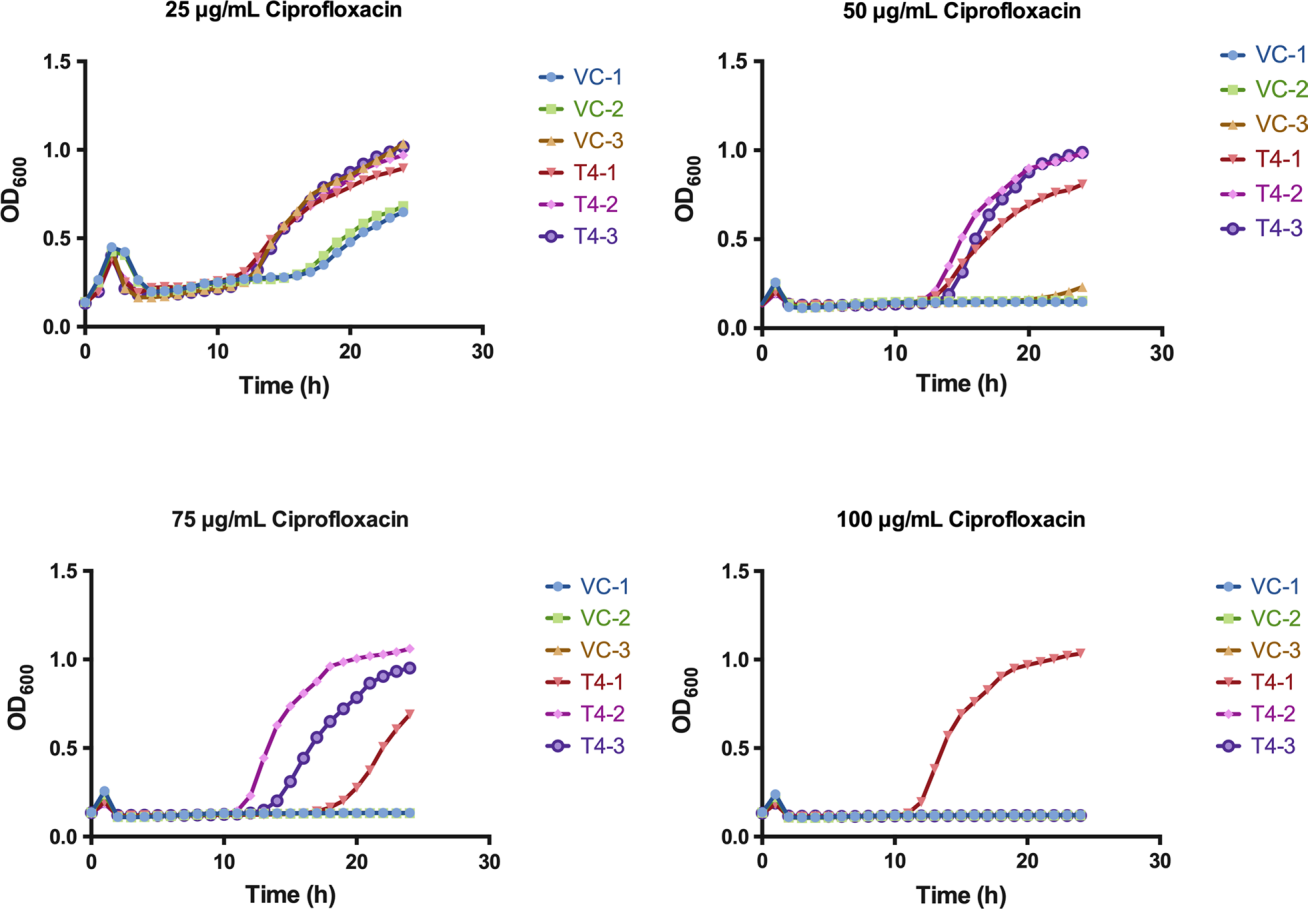
T4 DNA ligase increases host cell survival to ciprofloxacin treatment. Growth curve of either *E. coli* (VC) or *E. coli* (T4) in presence of 25, 50, 75 and 100 μg/mL ciprofloxacin respectively (Y-axis: OD_600_; X-axis time (h)). Experiments were performed in triplicates

In an initial investigation, we found that T4 DNA ligase in vivo mediates the repair of DNA double breaks in an error and prone manner (Manuscript accepted). In the meantime, we did not observe any physiological change of the host when T4 DNA ligase was over-expressed in *E. coli*. To see if the genotoxic drugs, which may kill the bacteria by inducing the DNA double breaks in vivo, may affect the cell growth, ciprofloxacin was adopted. As shown in Fig. 1, both *E. coli* (VC) and *E. coli* (T4) strains can grow normally at 25 μg/mL ciprofloxacin. When ciprofloxacin increased to 50 μg/mL, wild type *E. coli* MG1655 cannot grow in liquid culture up to 24 h incubation, but the growth of *E. coli* (T4) was detected after 14 h, indicating that ciprofloxacin resistant mutants arising. In presence of 75 μg/mL and 100 μg/mL of ciprofloxacin, growth was also detected after about 14–20 h incubation in T4 ligase expressed strain (Fig. 1). Since ciprofloxacin causes chromosome DNA DSBs through inhibiting DNA gyrase and topoisomerase IV [27], this observation clearly indicated that expression of T4 DNA ligase in vivo contributes to chromosomal DNA repair, possibly DSBs repair, which in turn increases cell survival. The increased survival further improved survival-coupled mutagenesis, among which resistant mutants arise on the chromosome ultimately.

### T4 DNA ligase in vivo increases cell resistance to ionizing radiation

**Fig. 2:**
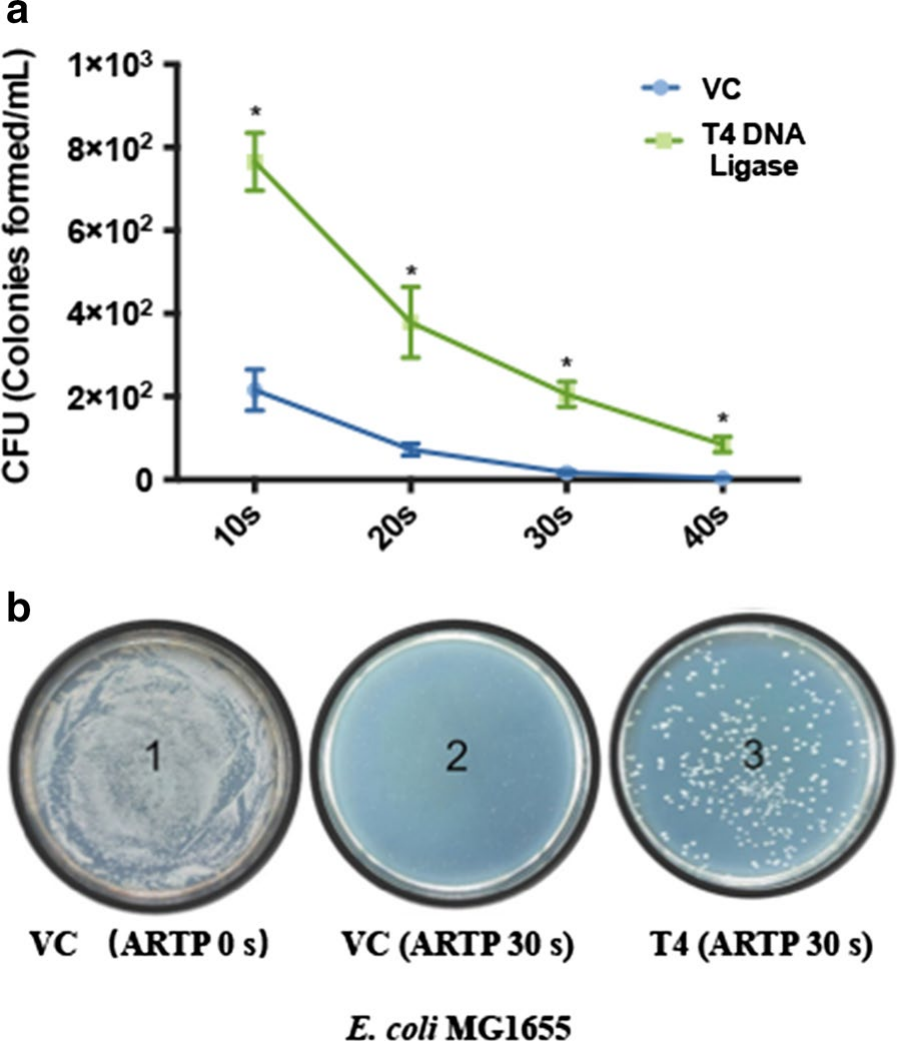
T4 DNA ligase confers cell survival to ionizing radiation (**a**). Survival of *E. coli* (VC) and *E. coli* (T4) to ARTP radiation. Equal number cells of *E. coli* MG1655 harboring empty vector (VC) or T4 DNA ligase expressing plasmid (T4 DNA ligase) were subjected to ARTP radiation for 10 s, 20 s, 30 s and 40 s, respectively. The resulting cells were plated onto agar plates to count CFU. An asterisk (*) stands for statistically significant difference (*p* < 0.001, unpaired *t-*test). **b** Cell survival of *E. coli* (VC) and *E. coli* (T4) upon ARTP exposure

To see if the cell expressing the T4 DNA ligase may also resistant to other DSBs causing factors, a new type of random mutagenesis machine ARTP, which causes various chromosomal damages including lethally DSBs, was employed [28]. To evaluate the contribution of T4 DNA ligase to host cell survival to ionizing radiation, cell survival of *E. coli* (VC) and *E. coli* (T4) was measured by CFU counts after ARTP exposure. As ionizing radiation time increased, survival of *E. coli* (VC) decreased correspondingly (Fig. 2a). After 30 s ARTP treatment, almost no survival was observed (Fig. 2a, b). Over increased ionizing radiation time, survival rate of *E. coli* (T4) dropped as vector control group did. Under same ionizing radiation time, however, *E. coli* (T4) showed a fivefold increase in survival to 10 s ARTP treatment in comparison to *E. coli* (VC) and cell survived up to 40 s ARTP treatment (Fig. 2a, b). This result clearly showed that T4 DNA ligase in vivo also increases the cell resistance to ionizing radiation, possibly via mediating the DSB repair caused by ARTP treatment.

### T4 DNA ligase mediated survival-coupled mutagenesis

Based on above results, a T4 mediated survival-coupled mutagenesis (T4SM) approach was proposed. When mutagens were used to mutagenize microbes for desired purpose, T4 DNA ligase can be employed and expressed in the host to repair chromosomal damages caused by mutagens. The host with repaired chromosome DSBs by T4 DNA ligase may survive for longer time and accumulate more mutations during the mutagen treatment. This will result in more mutations in one strain and a large survival pool for screening during the mutagenesis process.

To explore the feasibility of T4 DNA ligase-mediated microbial mutagenesis, the polyhydroxybutyrate (PHB) producing *E. coli* XLPHB, which harbors chromosomal integrated PHB biosynthesis genes *phbCAB* from *Ralstonia eutropha*, was subjected to mutagenesis and screening with T4 DNA ligase expressed in vivo [12]*.* Generally, there was nearly no colony on the plate after the cells were treated by ARTP for 60-s (Additional file 1: Fig. S1). However, expression of T4 DNA ligase in XLPHB increased host cell survival to 5 s ARTP radiation by five to sixfold (Additional file 1: Fig. S1a). Extended ARTP treatment obtained even more obvious result. This result proved the effectiveness of the T4SM method: the presence of T4 DNA ligase increased the survival rate and provided more mutated strains for further screening.

**Fig. 3:**
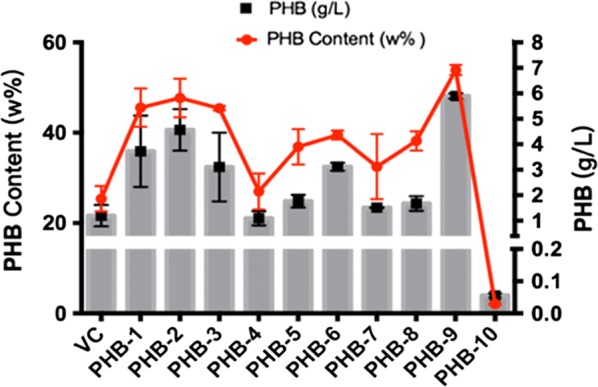
PHB fermentation results of *E. coli* XLPHB and ten randomly selected T4SM mutants

To estimate mutants acquired, ten colonies were then randomly selected and analyzed for their PHB production. As shown in Fig. 3, the control strain *E. coli* XLPHB accumulated 25.36 ± 2.82% w/w of the cell dry weight PHB. Mutants obtained by ARTP treatment vary dramatically in terms of PHB content. The highest PHB content was found to accumulate 112% more in comparison with the control and reached 53.87 ± 1.11% w/w of the cell dry weight. It indicates the methodology produces a library of mutants with diverse phenotypes.

**Fig. 4:**
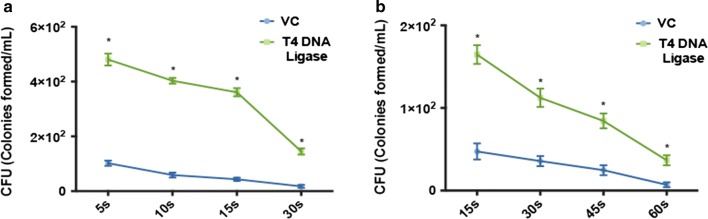
T4 mediated survival-coupled mutagenesis (T4SM) is applicable to *P. putida* and *L. plantarum.* **a** Survival of *P. putida* to ARTP radiation. Equal number cells of *P. putida* (VC) or *P. putida* (T4) were subjected to ARTP radiation for 5 s, 10 s, 15 s and 30 s respectively. The resulting cells were plated onto agar plates to count CFU. **b** Survival of *L. plantarum* to ARTP radiation. Equal number cells of *L. plantarum* (VC) or *L. plantarum* (T4) were subjected to ARTP radiation for 15 s, 30 s, 45 s and 60 s respectively. The resulting cells were plated onto agar plates to count CFU. Data shown are representative of three replicates and standard deviations were presented as error bars. An asterisk (*) stands for statistically significant difference (*p* < 0.001, unpaired *t-*test)

Two of the obtained mutants PHB-6 and PHB-10 with increased or decreased PHB accumulation were randomly selected and sent for draft genome sequencing. The results revealed both DNA fragment deletion and single nucleotide variation (SNV) mutation after ARTP treatment (Table 1). For mutant PHB-6, a 13-bp deletion was introduced inside the open reading frame of *yieK* gene, inactivating the putative 6-phosphogluconolactonase it encodes for. Inactivation of 6-phosphogluconolactonase may lead to the accumulation of 6-phosphogluconalactone, the product of glucose-6-phosphate oxidation and increase the supply of glucose-6-phospahte for glycolysis. Correspondingly, PHB-6 with mutated *yieK* increased PHB content from 25.36 ± 2.82% w/w to 39.47 ± 1.00%. This indicates that *yieK* is a novel target that has not been investigated and can potentially increase the carbon flux to glycolysis. In another sequenced mutant PHB-10, a 65 bp deletion residing in the ORF of *ilvB* gene, which encodes the acetoacetate synthase I subunit was found [29]. In addition, there was a 1-bp deletion between two ArcA-regulated repression sites upstream of *sdhC* promoter in this mutant [30]. Mutation of this site may interfere with the transcription of succinate dehydrogenase, the major component of the respiration chain. The very low cell mass of 2.72 ± 0.49 g/L and little PHB accumulation (1.95 ± 0.14%) of this mutant confirmed this prediction and draft sequencing result (Table 1). All these suggested that T4SM could greatly accelerate microbial breeding process by providing large mutants library. Mutants acquired by this approach bear mutation of SNV and short deletions.

**Table 1:** Identified mutations of PHB-6 and PHB-10

Mutation strain	Start site	Mutation	Gene	Product
PHB-6	3898712	Δ13 bp	*yieK*	Putative 6 phosphogluconolactonase
PHB-10	755154	Δ1 bp	Intergenic region of *gltA* and *sdhC*	Citrate synthase/succinate dehydrogenase subunit
	2080933	C→T	*sbmC*	DNA gyrase inhibitor
	3852572	Δ65 bp	*ilvB*	Acetolactate synthase 2 large subunit

### T4 DNA ligase mediated survival-coupled mutagenesis is applicable to both Gram-positive and Gram-negative bacteria

Contribution of T4 DNA ligase to host cell survival was also tested in Gram-negative bacterium *P. putida* and gram-positive bacterium *L. plantarum*. In *P. putida*, cell survival rate dropped correspondingly over time with radiation treatment (Fig. 4a). When T4 DNA ligase was expressed, the *P. putida* (T4) showed fivefold increased survival to ARTP treatment compared to that of *P. putida* (VC). Similar pattern was also observed in *L. plantarum*, in which T4 DNA ligase expression (*L. plantarum* (T4)) showed a fourfold increase after ARTP treatment compared to the control *L. plantarum* (VC) (Fig. 4b). These results suggested that T4 DNA ligase mediated survival to irradiation is applicable to wide range of bacteria either Gram-negative or Gram-positive and could be valuable for improving the random mutagenesis efficiency of these bacteria.

## Conclusion

In this study, we demonstrated that expression of T4 DNA ligase in vivo increase host cell survival to DSBs causing factors, such as genotoxic drugs and ionizing radiation. Based on this, T4 mediated survival-coupled mutagenesis (T4SM) was proposed, of which the effectiveness was validated by rapidly improving the PHB production in *E. coli* using ARTP treatment. Combined with high throughput screening methodology, the obtained mutants by the T4 mediated survival-coupled mutagenesis (T4SM) can now be sequenced to gain insights to the links between phenotypes and genomic variations. This indicated that traditional random mutagenesis shall be more powerful in combine with the modern genetic molecular biology and has exciting prospect.

## Materials and methods

### Bacterial strains and culture conditions

All bacteria strains are lab stocks and listed in Table 2. All *E. coli* and *P. putida* S16 strains were routinely cultured in Luria–Bertani (LB) broth with aeration at 220 rpm at 37 °C or 30 °C as indicated. The *L. plantarum* WCFS1 was routinely cultured in deMan Rogosa Sharpe (MRS) broth without aeration at stationary culture at 37 °C. Antibiotics were added to the following concentration when needed: spectinomycin (50 μg/mL), kanamycin (50 mg/mL) and erythromycin (250 μg/mL for *E. coli* and 25 μg/mL for *L. plantarum*). *E. coli* DH5α strain was used for molecular cloning and plasmids propagation. Engineered PHB producing *E. coli* XLPHB strain was subjected to ARTP treatment [12, 31].

**Table 2:** Bacterial strains and plasmids used in the study

Strain	Description	Source or reference
Bacterial strains		
* E. coli* MG1655	F^−^, lambda^−^, *rph*-1	[32]
* E. coli* XLPHB	DH5α*ΔpoxB::*M*(p5tac-phbCAB-kan)*	[12]
* Pseudomonas putida* S16	Wild type	[33]
* Lactobacillus plantarum* WCFS1	Wild type	[34]
* E. coli* DH5α	F^–^ φ80*lac*ZΔM15 Δ(*lac*ZYA-*arg*F)U169 *rec*A1 *end*A1 *hsd*R17(r_K_^–^, m_K_^+^) *pho*A *sup*E44 λ^–^ *thi*-1 *gyr*A96 *rel*A1	Invitrogen
* E. coli* (VC)	*E. coli* MG1655 strain harboring plasmid pUCLR4	This work
* E. coli* (T4)	*E. coli* MG1655 strain harboring plasmid pUCLR4-T4	This work
XLPHB (VC)	*E. coli* XLPHB strain harboring plasmid pUCLR4	This work
XLPHB (T4)	*E. coli* XLPHB strain harboring plasmid pUCLR4-T4	This work
* P. putida* (VC)	*P. putida* S16 strain harboring plasmid pBBR1MCS-2	This work
* P. putida* (T4)	*P. putida* S16 strain harboring plasmid pBBR-T4	This work
* L. plantarum* (VC)	*L. plantarum* WCFS1 strain harboring plasmid pE	This work
* L. plantarum* (T4)	*L. plantarum* WCFS1 strain harboring plasmid pE-T4	This work
Plasmids		
p15A-L4	Low copy number cloning vector, P15A origin of replication	[35]
pUCLR4	High copy number cloning vector, pUC origin of replication, Spe^R^,	This work
pUCLR4-T4	pUCLR4 derivative, T4 DNA ligase expressed from a constitutive PJ23104 promoter	This work
pBBR1MCS-2	Broad host range cloning vector, pBBR1 origin of replication, Kan^R^, low copy number	[33]
pBBR-T4	pBBR1MCS-2 derivative; T4 DNA ligase gene, expressed from a constitutive PJ23104 promoter	This work
pE	*E. coli*-*L. plantarum* shuttle vector, pUC origin of replication, 256 origin of replication (low copy number)_,_ Erm^R^	[36]
pE-T4	pE derivative, T4 DNA ligase gene, expressed from a constitutive PJ23104 promoter	This work

### Plasmid construction

All plasmid used in this study are listed in Table 2. All primers used in this study are listed in Additional file 1: Table S1. The pUCLR4 plasmid was assembled from the LR4 spacer amplified from previously reported plasmid p15A-L4 [35] using primers gRNA Spc-F/gRNA Spc-R, the pLtet promoter amplified from pwtCas9 plasmid using pLtet-F/pLtet-R and pUC Ori amplified from pUC19 plasmid using Ori-F/Ori-R by Gibson assembly [37].

Plasmid pUCLR4-T4 was assembled from T4 DNA ligase gene amplified from Enterobacteria phage T4 using primers T4-F/T4-R and pUCLR4 backbone amplified using primers T4 (ori terminator)-F/Ori (terminator)-R by Gibson assembly [37]. T4 DNA ligase gene was cloned under the control of a constitutive PJ23104 promoter (https://parts.igem.org/Part:BBa_K1468000).

To express T4 DNA ligase in *P. putida* S16, the T4 DNA ligase gene with an upstream constitutive PJ23104 promoter was amplified from pUCLR-T4 plasmid using primers T4 HindIII-F/T4 KpnI-R and cloned into vector pBBR1MCS-2 at *Hin*dIII and *Kpn*I sites, resulting in plasmid pBBR-T4.

To express T4 DNA ligase in *L. plantarum,* plasmid pE-T4 was assembled from the T4 DNA ligase gene with an upstream constitutive PJ23104 promoter amplified from pUCLR-T4 plasmid using primers T4 pE-F/T4 pE-R and the pE plasmid backbone amplified using primers pE T4-F/pE T4-R by Gibson assembly [37].

### Bacterial transformation

*Escherichia coli* transformation was performed by chemical transformation. Transformation of *L. plantarum* by electroporation was performed as previous described [38]. Briefly, *L. plantarum* cells cultured to mid-exponential phase (OD_600_ ~ 0.4–0.6) were collected, washed twice with SM buffer (952 mM sucrose supplemented with 3.5 mM MgCl_2_) and resuspended in SM buffer. Plasmid DNA ( ~ 1 μg) was added to 100 μL prepared competent cells. The resulting cell mixture were incubated on ice for 10 min. Cell mixture were electroporated using 2 mm electroporation cuvette and Gene Pulser (BioRad) under following condition: 2000 V, 25 μF, 400 Ω. Cells was recovered in SMRS broth (MRS broth supplemented with 0.5 M sucrose and 0.1 M MgCl_2_) at 37 °C for 3 h before spreading on MRS agar plates containing erythromycin.

Transformation of *P. putida* by electroporation following previously published protocol [39]. *P. putida* cells cultured to mid- exponential phase (OD_600_ ~ 0.2–0.4) were collected, washed twice with 300 mM sucrose solution and resuspended in 300 mM sucrose. After 10 min incubation on ice, the mixture of competent cells and DNA ( ~ 1 μg) was electroporated using 2 mm electroporation cuvette and Gene Pulser (BioRad) under following condition: 2500 V, 25 μF, 400 Ω. Cells was recovered in LB broth at 30 °C for 2 h before spreading on LB agar plates containing kanamycin.

### Ciprofloxacin sensitivity assay

Overnight culture of *E. coli* MG1655 harboring either plasmid pUCLR4 or pUCLR4-T4 was sub-cultured to fresh LB medium supplemented with spectinomycin. Subcultures were transferred to 24-well microtiter plate. Ciprofloxacin was added to cultures to final concentrations of 25 μg/mL, 50 μg/mL, 75 μg/mL or 100 μg/mL, respectively. Growth was monitored by measuring OD_600_ every hour for 48 h using plate reader (BioTek Synergy HT) at 37 °C with shaking.

### Atmospheric and room temperature plasma treatment

Bacterial strains were collected when grown to mid-log phase and washed by NaCl solution (normal saline) twice. Equal number of cells were re-suspended in NaCl solution to a standard OD_600_ (1.0). For each sample, 10 μL of the resulting cultures was spread onto stainless disc. The sample disc was placed 2 mm below the plasma torch nozzle exit. All ARTP irradiation treatments were performed using atmospheric and room temperature plasma (ARTPII, Tmaxtree Biotechnology) with radiofrequency power input at 100 W and gas flow at 10 SLM (standard liters per minute) at room temperature (15–30 °C). Immediately after the radiation, 1 mL of NaCl solution was added to the sample disc to re-suspend bacteria by vortex. CFU was counted for the resulting samples. Samples treated the same while lacking radiation was plated and analyzed as control groups.

### PHB fermentation and content determination

Sample of *E. coli* XLPHB treated with ARTP was plated onto CongoRed agar to identify PHB producers. Single colony of each mutant tested was inoculated into 5 mL LB medium and cultured for 12 h at 37 °C with aeration at 180 rpm. Cultures were sub-cultured at 4:100 ratio to fresh 50 mL LB medium with 30 g/L glucose at 37 °C with aeration at 180 rpm. Cells were collected after 48 h of fermentation and freeze-dried for 12 h (the weight of total dried cells is measured as Mx). PHB content of 10–20 mg (mx) freeze-dried cells were measured by gas chromatography after methanolysis as previously described [40]. Collected dried cells for PHB content analysis was weight as mx. The PHB content was calculated as m(PHB)/mx (stands for content of PHB per mg dried cells); total production of PHB was calculated as m(PHB) * Mx/mx.

### Genome DNA sequencing and analysis

Genome DNA of desired sample was extracted using TIANamp Bacteria DNA Kit (Tiangen, China) and sequenced by GENEWIZ. The resequencing method was used for the analysis of single nucleotide variant (SNV), insertion and deletion (INDEL) as well as structure variant. Genome of *E. coli* MG1655 was used as the reference genome (https://www.ncbi.nlm.nih.gov/nuccore/NC_000913.3).

### Statistical analysis

All experiments were performed three times in triplicates. Data shown were representative of three biological replicates with standard deviation as error bars.

## Additional file

**Additional file 1: Table S1.** Primers used in this study.

## Data Availability

Not applicable.

## Abbreviations

ARTP: atmospheric and room temperature plasma
PHB: polyhydroxybutyrate
DSB: double strand break
