# Supplementary material for: The phage T4 DNA ligase in vivo improves the survival-coupled bacterial mutagenesis
Source: Microb Cell Fact. 2019 Jun 13;18:107. doi: 10.1186/s12934-019-1160-7 (PMC6567493; doi:10.1186/s12934-019-1160-7)
Supplement: Supplementary file 1 — Additional file 1: Table S1. Primers used in this study. [file 12934_2019_1160_MOESM1_ESM.docx]

Additional Table S1

| Primer name | Primer sequence（5’-3’） |
| --- | --- |
| gRNA Spc-F | ACTAGTTGAGACCAGTCTCGGAAG |
| gRNA-Spc-R | ACGCTCTCCATAAGCCTGTTCGGTTCGTAAGC |
| pLtet-F | CTTAAGACCCACTTTCACATTTAAG |
| pLtet-R | GACGTCTCCCTAGGTATAAACGCAG |
| ori-F | GCTAGCACAATACCTAGGACTGAGCTAGCTGTCAAAGGCGGTAATACGGTTATCCACA |
| ori-R | ACGCTCTCCACTGAGCGTCAGACCCCGTAG |
| T4-F | CTGACGCTCAGTGGAGAGCGTGCTTTCATAGACCAGTTACCTCATG |
| T4-R | CAGTCCTAGGTATTGTGCTAGCGTTAGAACCACGTACCACAGG |
| T4(ori terminator)-F | TCATGAGGTAACTGGTCTATGACCATGGGAATTCAGATCTCCTCG |
| Ori(terminator)-R | GGCGGTAATACGGTTATCCACAGAATCAGGGGATAACGCAGGAAA |
| T4 HindIII-F | CCCAAGCTTGAGAGCGTGCTTTCATAGACCAGTT |
| T4 KpnI-R | CGGGGTACCTTGACAGCTAGCTCAGTCCTAGGTA |
| T4 pE-F | AAAGCAATTACTGATACGTTACCACCGCTGCGTTCGGTC |
| T4 pE-R | TTCTGCTCCCGCCCTTAGCTCACATGTTCTTTCCTGCGT |
| pE T4-F | GAAAGAACATGTGAGCTAAGGGCGGGAGCAGAATGTCCG |
| pE T4-R | CGCAGCGGTGGTAACGTATCAGTAATTGCTTTATCAACT |

Additional Figure S1


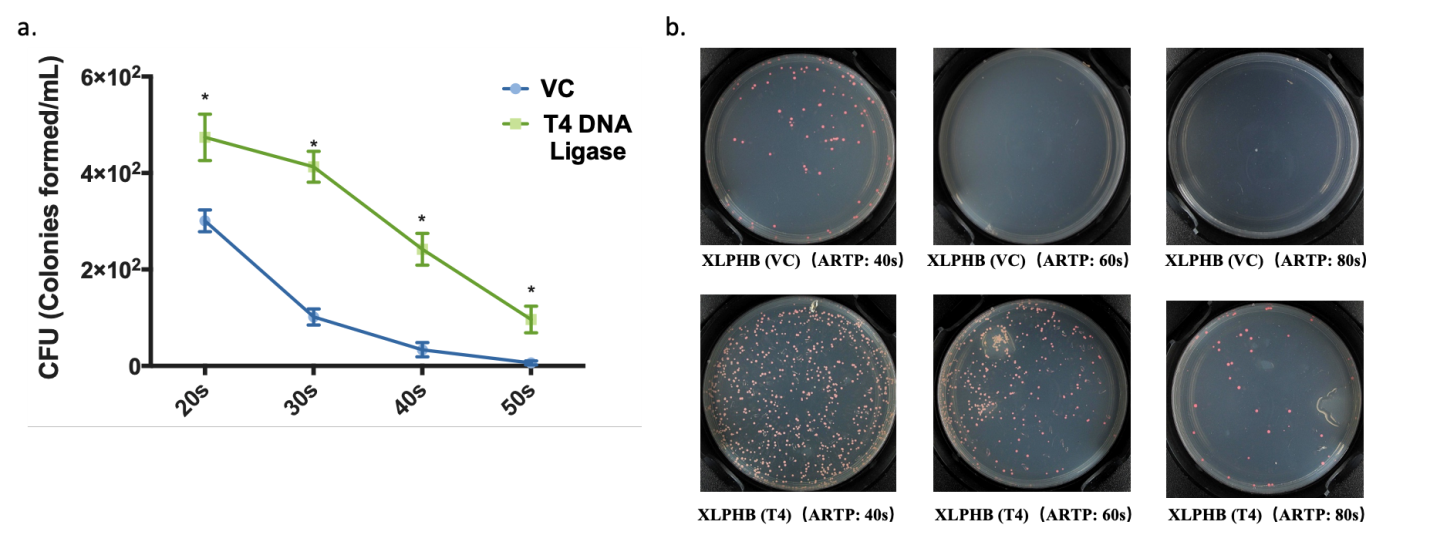


**Figure S1 T4-mediated survival-coupled mutagenesis in PHB producing strain *E. coli* XLPHB-T4**. **(A)** Survival of *E. coli* XLPHB (T4) or XLPHB(VC) to ARTP radiation for 20 sec, 30 sec, 40 sec and 50 sec, respectively. An asterisk (*) stands for statistically significant difference (*p*<0.001, unpaired *t-*test) (B) results of *E. coli* XLPHB (T4) under different atmospheric and room temperature plasma (ARTP) treatment time conditions (40s, 60s, 80s).
